# Supplementary material for: A SET domain-containing protein and HCF-1 maintain transgenerational epigenetic memory
Source: Nat Commun. 2026 Jan 9;17:1462. doi: 10.1038/s41467-025-68200-7 (PMC12886896; doi:10.1038/s41467-025-68200-7)
Supplement: Supplementary file 1 — Supplementary Information [file 41467_2025_68200_MOESM1_ESM.pdf]

Supplementary information for Zeng et al.

A SET domain-containing protein and HCF-1 maintain transgenerational epigenetic memory

Chenming Zeng[1,2,#], Giulia Furlan[2,#], Miguel Vasconcelos Almeida[1,2,#], Juan C. Rueda-Silva[1,2,3], Jonathan Price[1,2], Helena Santos-Rosa[1,2], Jingxiu Xu[1], Yan Kuang[4], Enric Cata Socias[1], Jonas Mars[2], Pedro Rebelo-Guiomar[1], Meng Huang[4], Shouhong Guang[4], Falk Butter[5,6], Eric A. Miska[1,2,3,7*]

1 Department of Biochemistry, University of Cambridge, Cambridge, CB2 1GA, United Kingdom

2 The Gurdon Institute, University of Cambridge, Cambridge, CB2 1QN, United Kingdom

3 Department of Genetics, University of Cambridge, Cambridge, CB2 3EH, United Kingdom

4 School of Life Science, University of Science and Technology of China, Hefei, Anhui, 230027, China

5 Institute of Molecular Biology, Mainz, 55128, Germany

6 Institute of Molecular Virology and Cell Biology, Friedrich-Loeffler-Institute, Greifswald, 17493, Germany

7 Wellcome Sanger Institute, Wellcome Trust Genome Campus, Cambridge, CB10 1SA, United Kingdom

# These authors contributed equally to this work.

* Corresponding author: eam29@cam.ac.uk

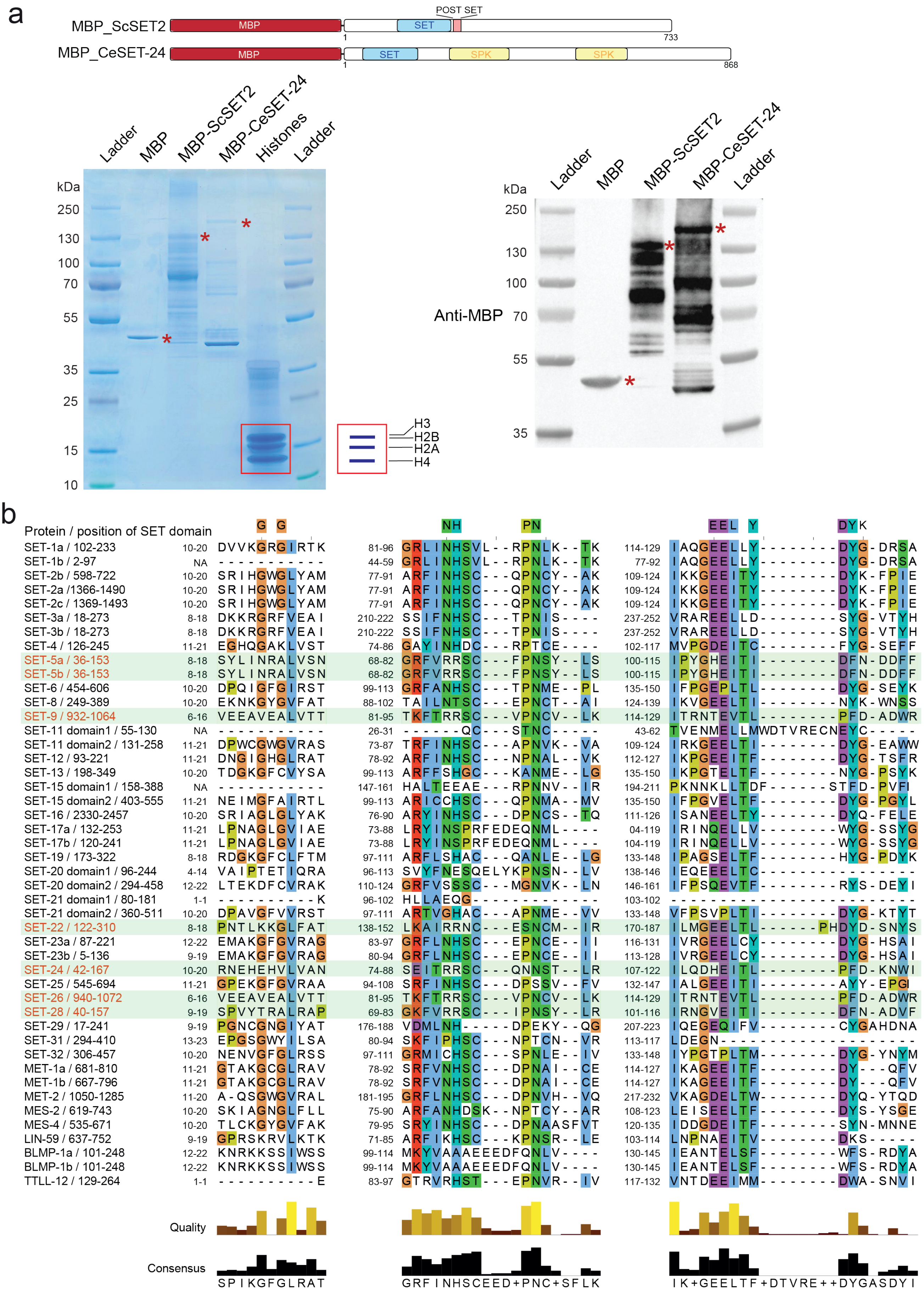

a
MBP_ScSET2
MBP
SET
POST SET
1
733
MBP_CeSET-24
MBP
SET
SPK
SPK
1
868
Ladder
MBP
MBP-ScSET2
MBP-CeSET-24
Histones
Ladder
kDa
250
130
100
70
55
35
25
15
10
H3
H2B
H2A
H4
Anti-MBP
b
Protein / position of SET domain
SET-1a / 102-233
SET-1b / 2-97
SET-2b / 598-722
SET-2a /1366-1490
SET-2c / 1369-1493
SET-3a / 18-273
SET-3b / 18-273
SET-4 / 126-245
SET-5a / 36-153
SET-5b / 36-153
SET-6 / 454-606
SET-8 / 249-389
SET-9 / 932-1064
SET-11 domain1 / 55-130
SET-11 domain2 / 131-258
SET-12 / 93-221
SET-13 / 198-349
SET-15 domain1 / 158-388
SET-15 domain2 / 403-555
SET-16 / 2330-2457
SET-17a / 132-253
SET-17b / 120-241
SET-19 / 173-322
SET-20 domain1 / 96-244
SET-20 domain2 / 294-458
SET-21 domain1 / 80-181
SET-21 domain2 / 360-511
SET-22 / 122-310
SET-23a / 87-221
SET-23b / 5-136
SET-24 / 42-167
SET-25 / 545-694
SET-26 / 940-1072
SET-28 / 40-157
SET-29 / 17-241
SET-31 / 294-410
SET-32 / 306-457
MET-1a / 681-810
MET-1b / 667-796
MET-2 / 1050-1285
MES-2 / 619-743
MES-4 / 535-671
LIN-59 / 637-752
BLMP-1a / 101-248
BLMP-1b / 101-248
TTLL-12 / 129-264
Quality
Consensus
SPIKGFGLRAT
GRFINHSCEED+PNC+SFLK
IK+GEELTF+DTVRE++DYGASDYI

Supplementary Fig. 1. Related to Figure 1. **Alignment of *C. elegans* SET domains.**
a Schematic of MBP-tagged ScSET2 (SET2 from *Saccharomyces cerevisiae*) and CeSET-24 (SET-24 from *Caenorhabditis elegans*). Coomassie blue staining of Calf Histones and purified MBP tagged ScSET2 and CeSET-24 (bottom left) and western blotting of purified MBP tagged proteins (bottom right, anti-MBP, ab9084) used in the Fig. 1e. MBP, Maltose-Binding Protein. Red stars indicate MBP-tagged proteins. The expected molecular weight of MBP-tagged proteins: MBP, 42 kDa; MBP-ScSET2, 132 kDa; MBP-CeSET-24, 145 kDa. *n* = 3 independent experiments with similar results. The uncropped and unprocessed version of the Western blot is provided in Source Data. **b** Multiple sequence alignment of SET domains. Residues critical for catalytic activity are highlighted. SET proteins in the branch highlighted in Fig. 1a are indicated with red labels and green background.

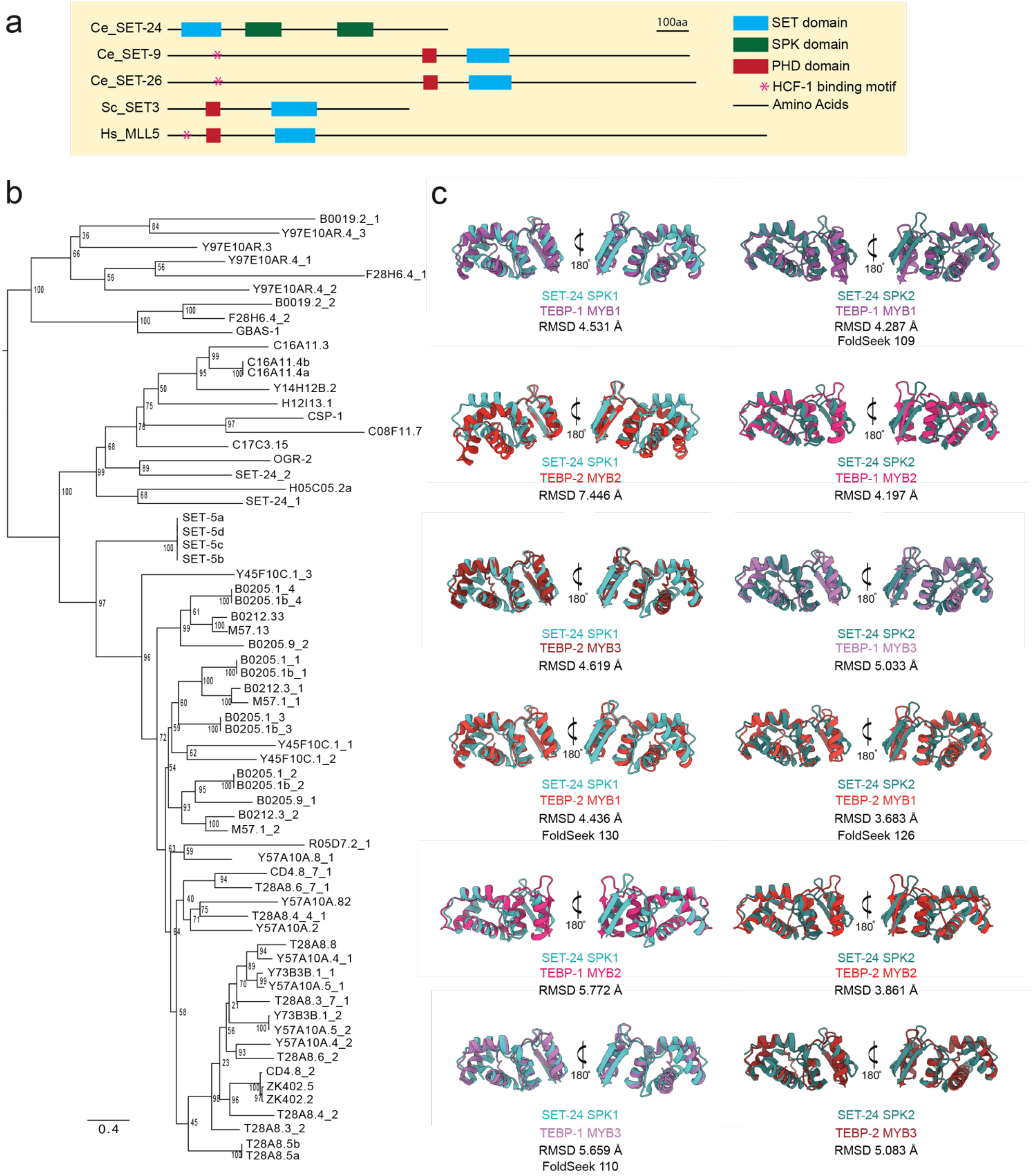

Supplementary Fig. 2. Related to Figure 1. **Evolutionary and structural analysis of SPK domains.**
a Schematic representation of sequences of CeSET-24 and ScSET3 subfamily proteins with domain information. **b** Maximum likelihood phylogenetic tree comparing the protein sequences of SPK domains in *C. elegans*. Values next to the tree nodes are branch supports, calculated with 1,000 ultrafast bootstrap replicates. **c** The structural alignments show the similarity between the SPK domain of SET-24 and the MYB domains of TEBP-1 and TEBP-2, as predicted by Alphafold3.

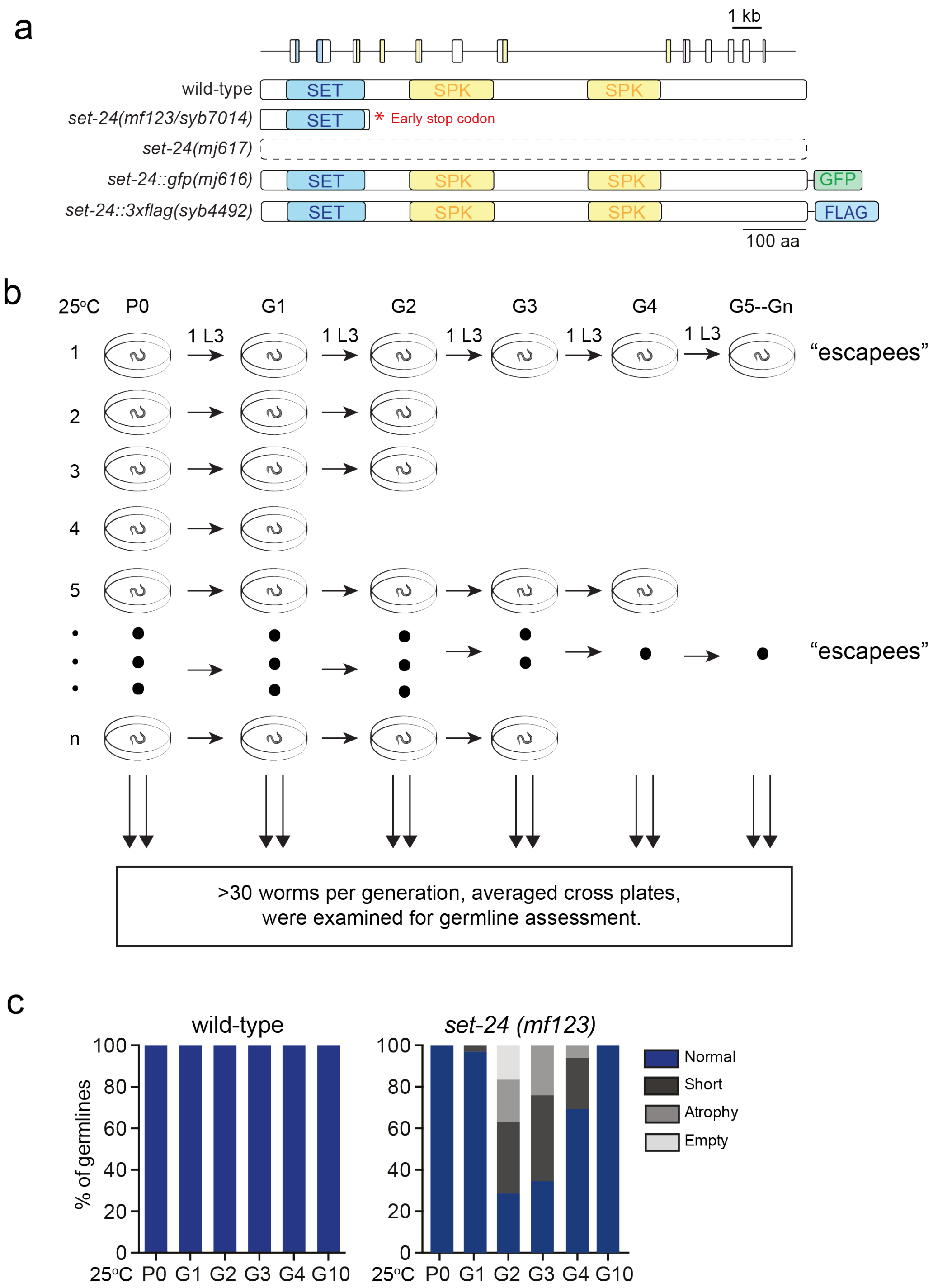

Supplementary Fig. 3. Related to Figure 2**. SET-24 is required for germline development.**
a Schematic representation of the *set-24* locus (drawn to scale) and the predicted protein products corresponding to the wild-type, mutant, and tagged alleles used in this study. **b** A scheme of Mrt assay procedure. Singled L3-stage worms were placed on HB101-seeded plates and maintained at 25 °C to grow and fertilize. For each subsequent generation, a single L3 worm was picked to serve as the parent of the next generation, continuing until sterility or the end of the experiment. At each generation, *n* > 30 worms were sampled evenly from independent lines for germline assessment. **c** *set-24(mf123)* mutant worms, the same mutation isolated from the wild strain display progressive germline degeneration. Proportions of normal, short, atrophic, and empty germlines (*n* > 30 worms per generation). Source data are provided as a Source Data file.

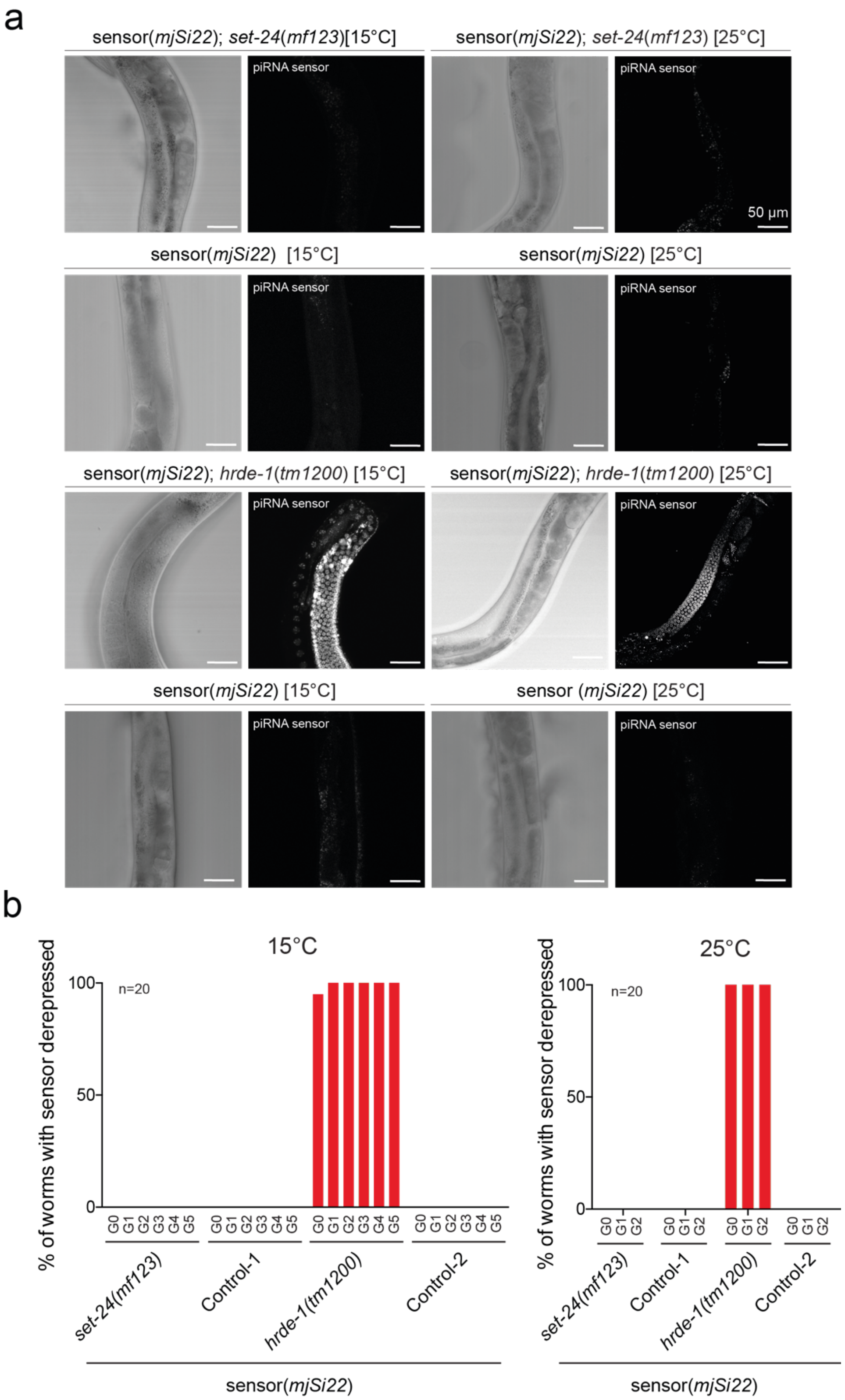

Supplementary Fig. 4. Related to Figure 3. ***set-24* is dispensable for initiation of piRNA-dependent silencing.**

a Representative images of G2 generation germlines of the indicated genotypes. **b** Quantification of the percentage of derepressed individuals at every generation (counting on *n* > 5 worms per generation). Source data are provided as a Source Data file.

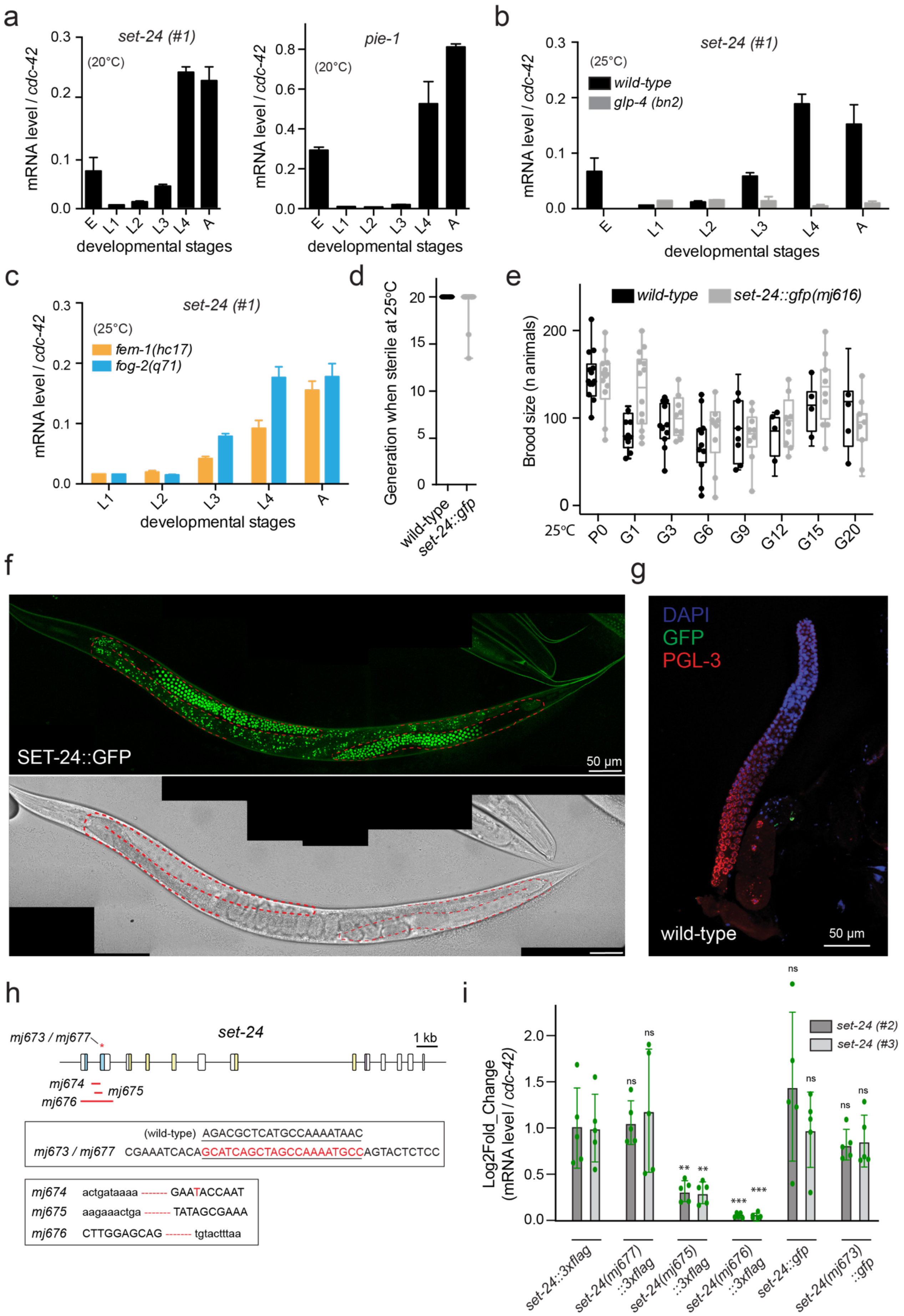

a
set-24 (#1)
(20°C)
mRNA level / cdc-42
developmental stages
E L1 L2 L3 L4 A
pie-1
(20°C)
b
set-24 (#1)
(25°C)
wild-type
glp-4 (bn2)
c
set-24 (#1)
(25°C)
fem-1(hc17)
fog-2(q71)
d
Generation when sterile at 25°C
wild-type
set-24::gfp
e
wild-type
set-24::gfp(mj616)
Brood size (n animals)
25°C P0 G1 G3 G6 G9 G12 G15 G20
f
SET-24::GFP
50 μm
g
DAPI
GFP
PGL-3
wild-type
50 μm
h
mj673 / mj677
set-24
1 kb
mj674
mj675
mj676
(wild-type) AGACGCTCATGCCAAAATAAC
mj673 / mj677 CGAAATCACAGCATCAGCTAGCCAAAATGCCAGTACTCTCC
mj674 actgataaaa ------- GAATACCAAT
mj675 aagaaactga ------- TATAGCGAAA
mj676 CTTGGAGCAG ------- tgtactttaa
i
Log2Fold_Change
(mRNA level / cdc-42)
set-24 (#2)
set-24 (#3)
ns
**
***
set-24::3xflag
set-24(mj677) ::3xflag
set-24(mj675) ::3xflag
set-24(mj676) ::3xflag
set-24::gfp
set-24(mj673) ::gfp

Supplementary Fig. 5. Related to Figure 4. **SET-24 is a germline-specific factor.**
a *set-24* is germline-specific. Developmental time-course analysis of *set-24* (left) and *pie-1* (right) expression by RT-qPCR in wild-type animals grown at 20ºC. Means and standard deviations are shown, *n* = 3 independent replicates. Source data are provided as a Source Data file. **b** and **c** RT-qPCR analysis of *set-24* expression in wild-type and germline-less *glp-4(bn2ts)* animals grown at 25 °C (**b**) and in masculinized *fem-1(hc17)* and feminized *fog-2(q71)* mutants grown at 25 °C (**c**). Means and standard deviations are shown, *n* = 3 independent replicates. Source data are provided as a Source Data file. **d** and **e** SET-24::GFP animals are not germline mortal at 25°C. Assessment of fertility, *n* = 15 worms (**d**) and quantification of the number of progeny over generations (P0, parental generation; G1-G20, generations 1 to 20; *n* = 15 worms per genotype, **e**). Source data are provided as a Source Data file. **f** Representative image of an adult worm expressing SET-24::GFP (top) and bright field (bottom). Red dashed lines indicate the germline. *n* = 10 independently imaged worms with similar results**. g** Representative immunofluorescence image of control wild-type animals stained with anti-GFP and anti-PGL-3 antibodies. Compare with Fig. 4a. *n* = 3 independently imaged worms with similar results. **h** Schematic representation of *set-24* mutations in Fig. 4e. **i** RT-qPCR analysis of *set-24* expression in the indicated strains. Two pairs of primers were used. Means and standard deviations are shown, *n* = 5 independent replicates, Stars indicate *p*-values, ns, not significant; **0.001 ≤ *p*-value < 0.01; ****p*-value <0.001. Comparisons were with *set-24::3xflag* strain. . The corresponding data points are shown. Source data and exact *p*-values are provided as a Source Data file.

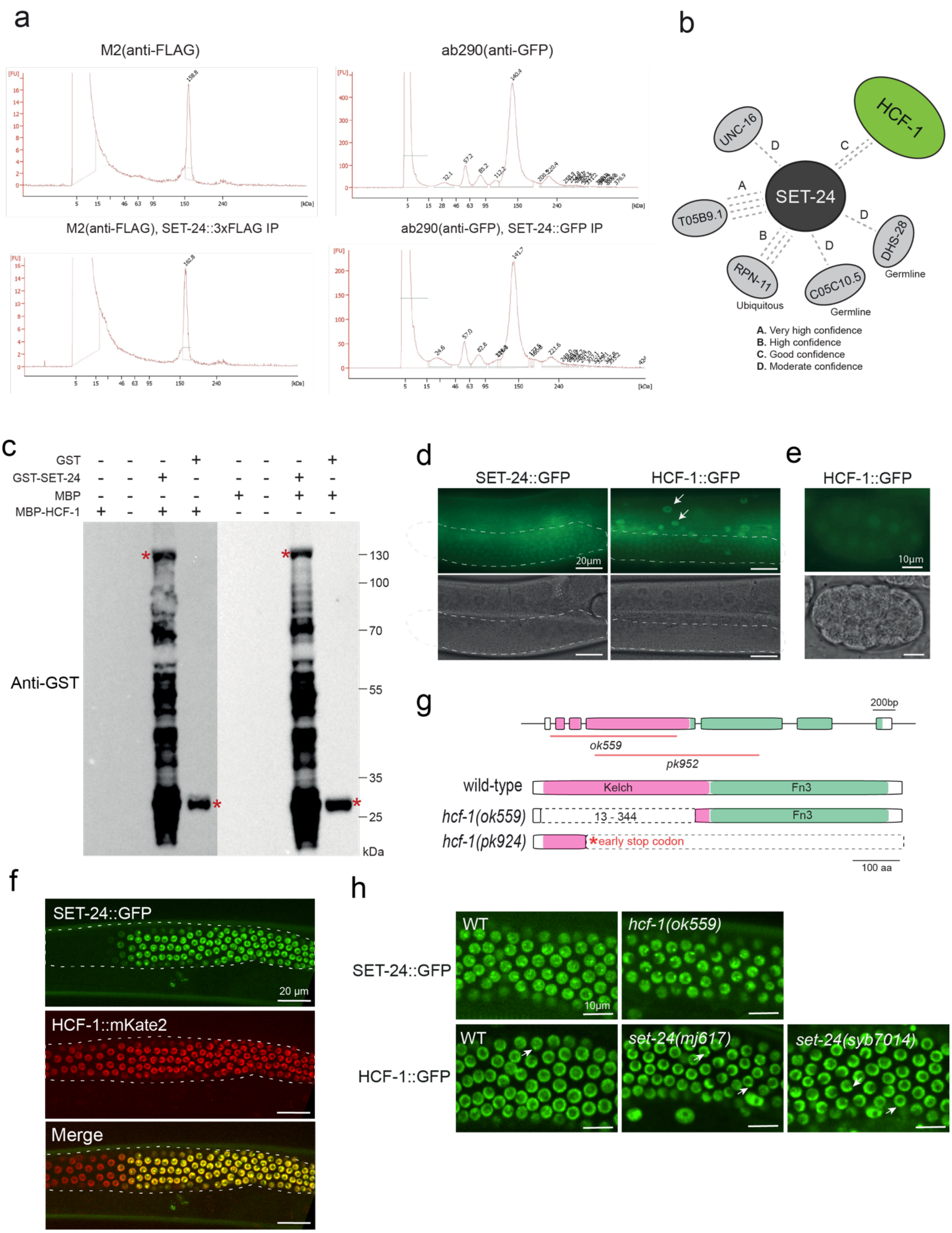

Supplementary Fig. 6. Related to Figure 5. **The expression patterns of SET-24 and HCF-1.**
a Profiles of proteins from SET-24::3xFLAG or SET-24::GFP ChIP and control, analysed using bioanalyzer. These profiles show that only antibodies, but not SET-24 and consequently no DNA for

sequencing, were recovered from the pull-downs. **b** Identification of SET-24 interactors via Yeast-two Hybrid. Panels "A-D" indicate the confidence levels of interaction. **c** Membranes shown in Fig. 5c were re-probed using anti-GST antibody (ab9085). Red stars indicate GST-tagged proteins. *n* = 3 independent experiments with similar results. **d** Representative images showing somatic expression of GFP-tagged SET-24 and HCF-1, marked by white narrows. The germline is marked by white dashed lines. *n* = 10 independently imaged worms with similar results**.** **e** A representative image showing the expression of GFP-tagged HCF-1 in the embryo. *n* = 10 independently imaged worms with similar results**.** **f** Representative images of *C. elegans* expressing SET-24::GFP and HCF-1::mKate2 in whole worms. White dashed lines indicate the germline. *n* = 10 independently imaged worms with similar results**.** **g** Schematic representation of the *hcf-1* locus (drawn to scale) and the predicted protein products corresponding to the wild-type and mutants used in this study. **h** Representative images of germlines of SET-24::GFP and HCF-1::GFP in wild-type and *hcf-1* or *set-24* mutant backgrounds. *n* = 10 independently imaged worms with similar results**.**

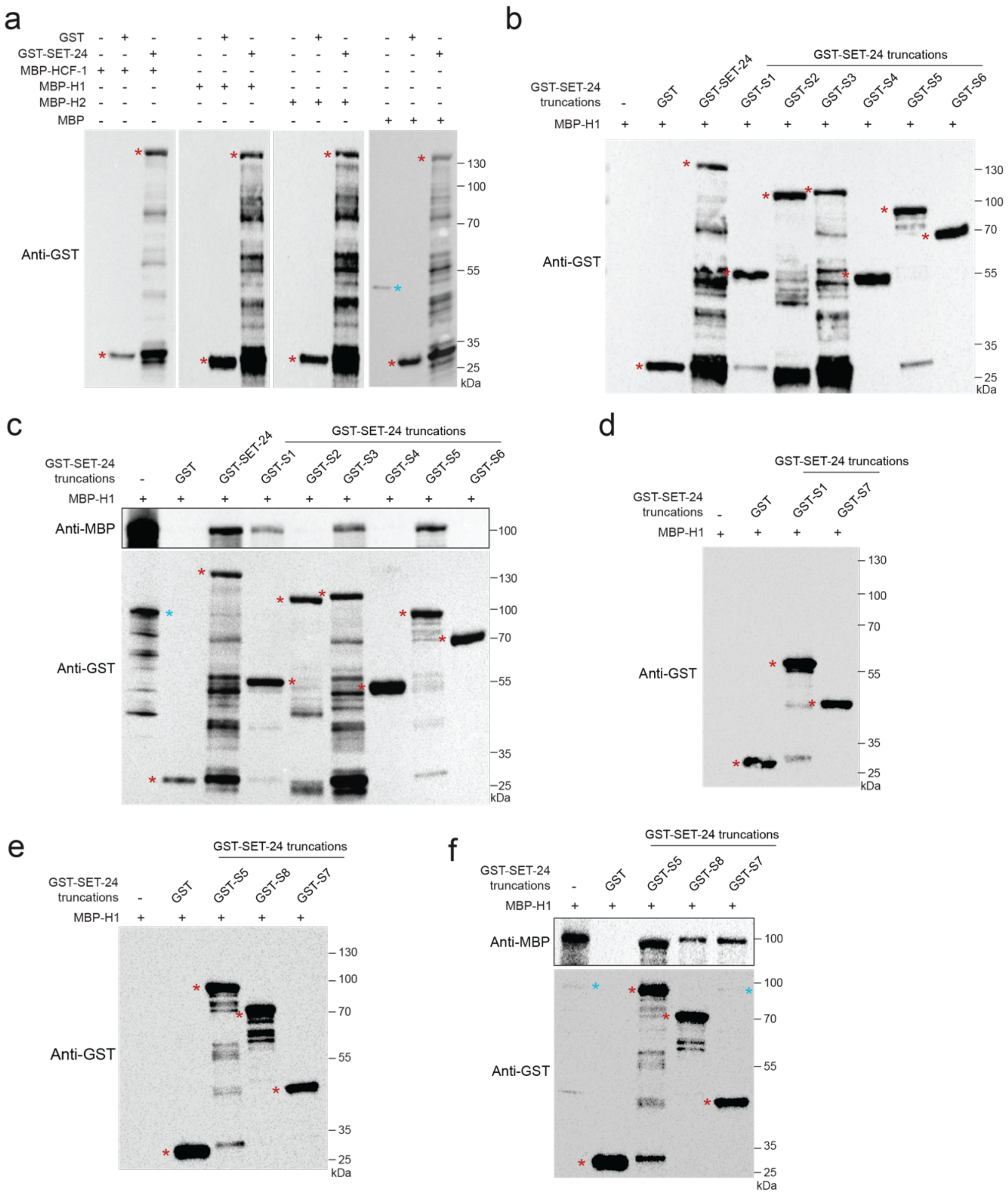

Supplementary Fig. 7. Related to Figure 5. **SET-24 and HCF-1 interact directly.**

a, **b, d, and e** Membranes shown in Fig. 5h (**a**), Fig. 5i (**b**), Fig. 5j (**d**), and Fig. 5k (**e**) were re-probed using an anti-GST antibody (ab9085). Red stars indicate GST-tagged proteins, and the blue star indicates a residual MBP band. *n* = 3 independent experiments with similar results. **c** and **f** *In vitro* GST-pulldown assays using GST-tagged SET-24 truncations to bind the MBP-tagged H1 fragment of HCF-1. Panels **c** and **f** are additional replicates of Figs. 5i, Supplementary Fig. 7b and 5k, Supplementary Fig. 7e, respectively, in which a different anti-MBP antibody was used (ab9084). The same membrane was then re-probed with an anti-GST antibody (ab9085). Red stars indicate GST-tagged proteins, and the blue stars indicates the residual MBP bands. *n* = 3 independent experiments with similar results. The uncropped and unprocessed versions of blots for (**a** - **f**) are provided in Source Data.

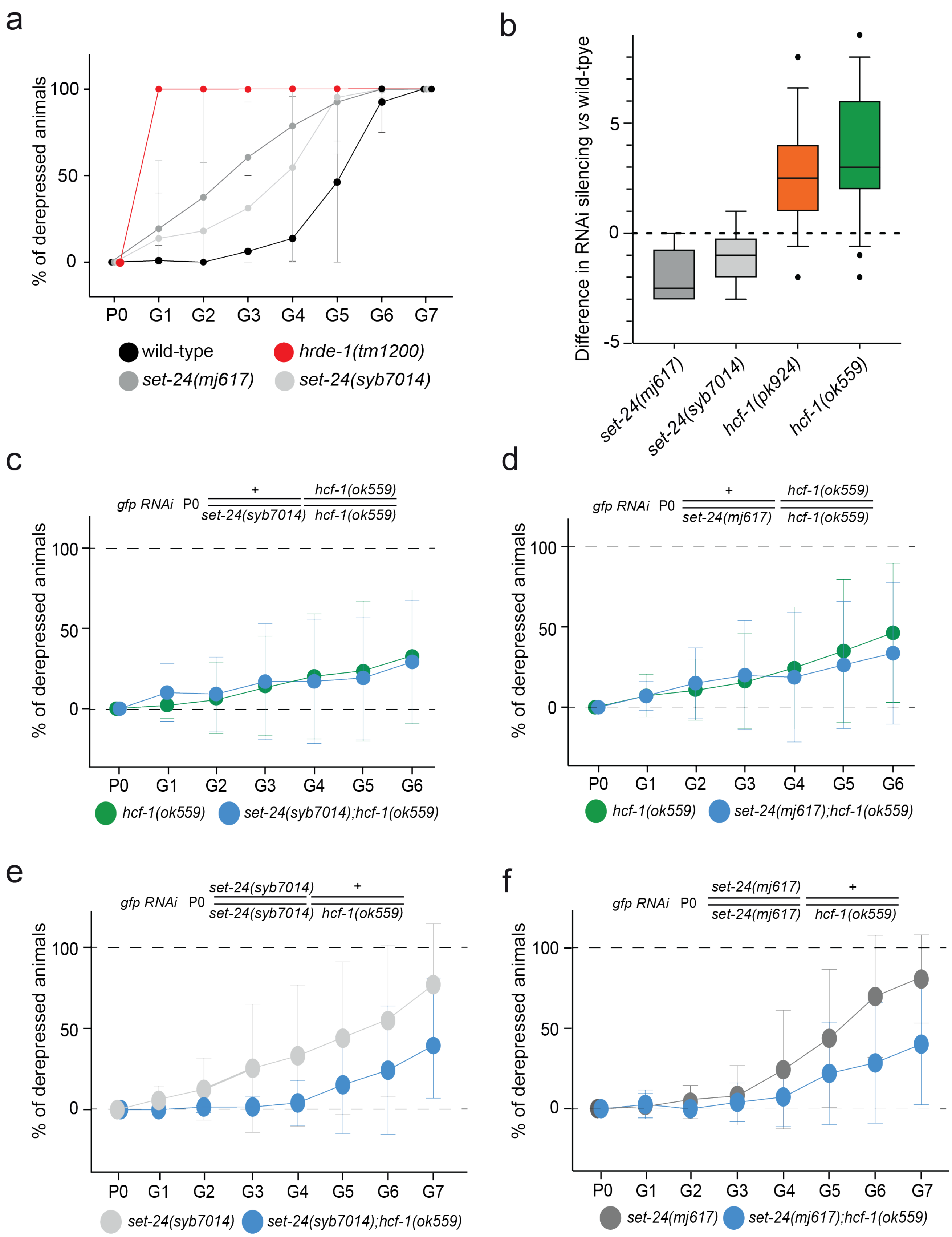

Supplementary Fig. 8. Related to Figure 6. **HCF-1 and SET-24 are required for the maintenance of heritable RNAi.**

a Quantification of the percentage of derepressed individuals at every generation. wild-type, *n* = 8 animal lineages; *hrde-1(tm1200)*, *n* = 5 animal lineages; *set-24(mj617)*, *n* = 4 animal lineages; *set-24(syb7014)*, *n* = 8 animal lineages. Source data are provided as a Source Data file. **b** The contrasts between generations of derepressed mutants and their corresponding wildtypes. The y-axis represents the difference in the number of generations of silencing between mutants and wildtypes. For instance, if silencing (defined as more than 50% of worms remaining silenced) continues until G8 in mutants but only until G5 in wildtypes, the contrast would be 3. Means and standard deviations are shown, *set-24(mj617)*, *n* = 6 contrasts; *set-24(syb7014)*, *n* = 8 contrasts; *hcf-1(pk924)*, *n* = 16 contrasts; *hcf-1(ok559)*, *n* = 23 contrasts. Source data are provided as a Source Data file. **c** - **f** Indicated individuals were fed with GFP RNAi bacteria. Silenced double and single mutants (all identified by genotyping PCR) were self-fertilized to produce the G1 and subsequent generations and were maintained on HB101-seeded plates. The percentage of derepressed individuals was quantified at each generation. Means and standard deviations are shown. (**c**) *hcf-1(ok559)*, *n* = 13 animal lineages, *set-24(syb7014); hcf-1(ok559)*, *n* = 12 animal lineages. (**d**) *hcf-1(ok559)*, *n* = 14 animal lineages, *set-24(mj617); hcf-1(ok559)*, *n* = 11 animal lineages. (**e**) *set-24(syb7014)*, *n* = 17 animal lineages, *set-24(syb7014); hcf-1(ok559)*, *n* = 13 animal lineages. (**f**) *set-24(mj617)*, *n* = 14 animal lineages, *set-24(syb7014); hcf-1(ok559)*, *n* = 11 animal lineages. Source data for (**a** - **f**) are provided as a Source Data file.

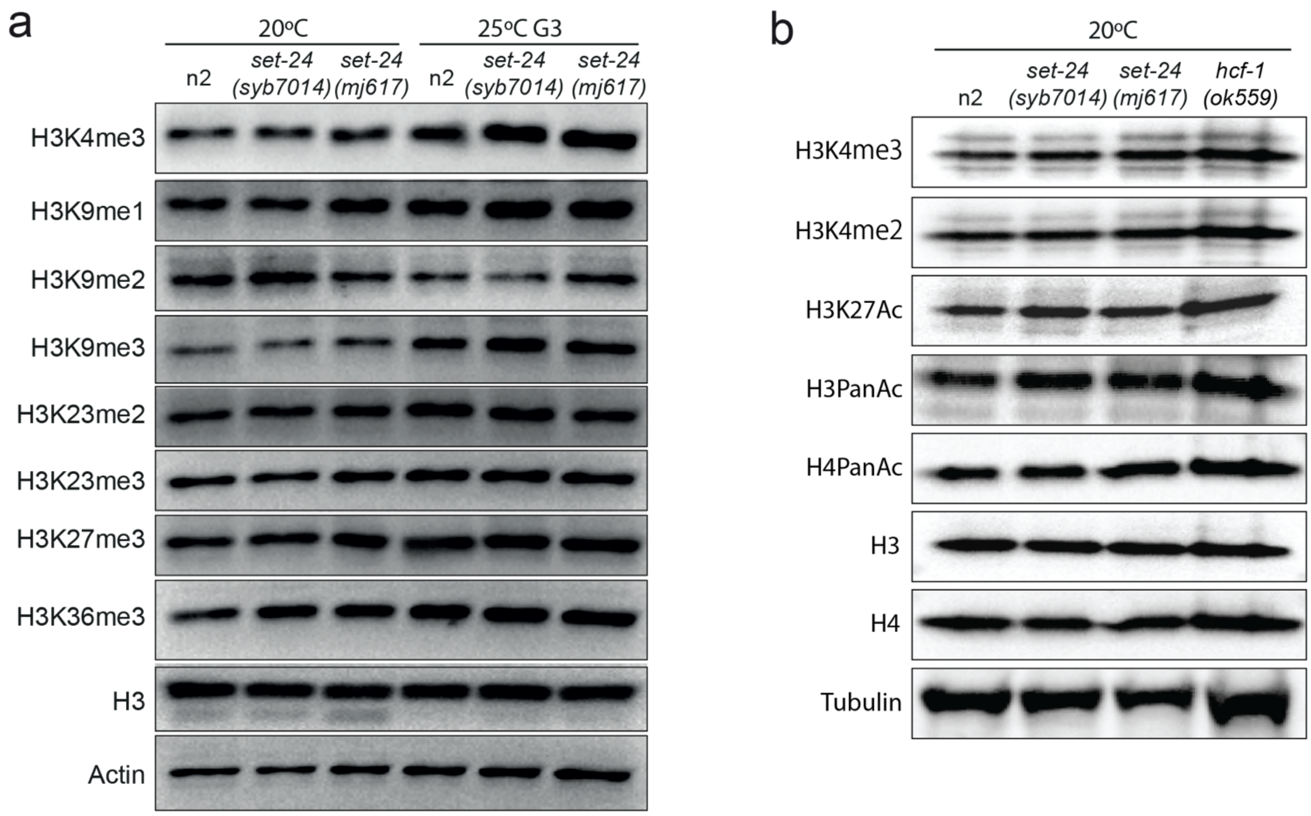

Supplementary Fig. 9. Related to Figure 7. **Western blot analysis of global histone methylation and acetylation levels in *set-24* and *hcf-1* mutants.**

a Western blot analysis of total extracts from wild-type and *set-24* mutant worms was performed using the specified antibodies. Worms were collected at the young adult stage, either grown at 20°C or after being cultured at 25°C for three generations. Antibodies: H3K4me3 (ab8580), H3K9me1 (ab9045), H3K9me2 (ab1220), H3K9me3 (07-523), H3K23me2 (39653), H3K23me3 (61499), H3K27me3 (07-449), and H3K36me3 (ab9050), H3 (ab1791), and β-actin (AF5003). **b** Western blot analysis of total extracts from wild-type, *set-24* and *hcf-1* mutant worms was performed using the specified antibodies. Worms were collected at the young adult stage grown at 20°C. Antibodies: H3K4me3(ab8580), H3K4me2(ab7766), H3K27Ac (ab4729), H3PanAc (ab47915, recognizing acetyl K9 + K14 + K23 + K23 + K27), H4PanAc (ab177790, recognizing acetyl K5 + K8 + K12 +K16), H3 (ab1791), H4 (ab10158), Tubulin (ab6160). The uncropped and unprocessed versions of blots for (**a** - **b**) are provided in Source Data.

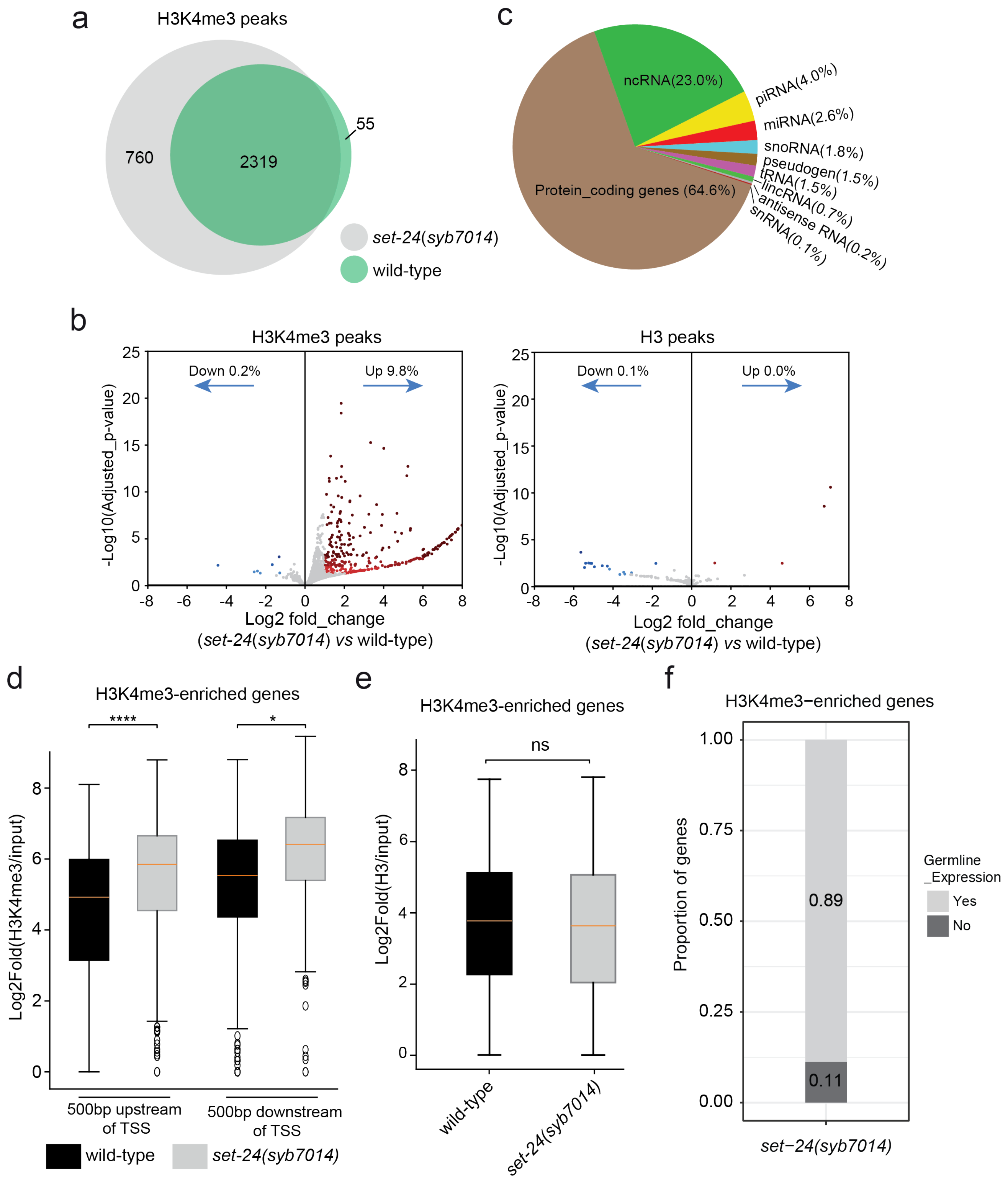

Supplementary Fig. 10. Related to Figure 7. **SET-24 modulates H3K4me3.**

a Venn diagram showing overlaps of H3K4me3 peaks between *set-24(syb7014)* and wild-type. **b** Volcano plots showing differentially enriched peaks of H3K4me3 (left) and H3 (right) in *set-24(syb7014)* compared to the wild-type. H3K4me3 ChIP-seq, two replicates each for *set-24(syb7014)* and wild-type; H3 ChIP-seq, three replicates each for *set-24(syb7014)* and wild-type. **c** Pie chart showing the genome features overlapping with upregulated H3K4me3 peaks in *set-24(syb7014)*. **d** Comparison of average H3K4me3 enrichment within 500 bp upstream and downstream of TSS for

wild-type samples and *set-24(syb7014)* mutants across H3K4me3-enriched genes with significant $\log_2$ fold-change. Statistical significance was assessed using a two-sided unpaired *t*-test. Asterisks indicate *p*-values: ****p-value < 0.001; *0.01 ≤ *p*-value < 0.05. The *p*-value for the comparison between wild-type and *set-24(syb7014)* at 500bp upstream of TSS is $2.48e^{-09}$, and the *p*-value for the comparison between wild-type and *set-24(syb7014)* at 500bp stream of TSS is 0.0037. **e** Comparison of the average H3 enrichment throughout the gene body for wild-type samples and *set-24*(*syb7014*) mutants across H3K4me3-enriched genes. Statistical significance was assessed using a two-sided unpaired *t*-test. ns, not significant. The *p*-value for the comparison between wild-type and *set-24(syb7014)* is 0.99. **f** Germline-expression analysis of H3K4me3-enriched genes.

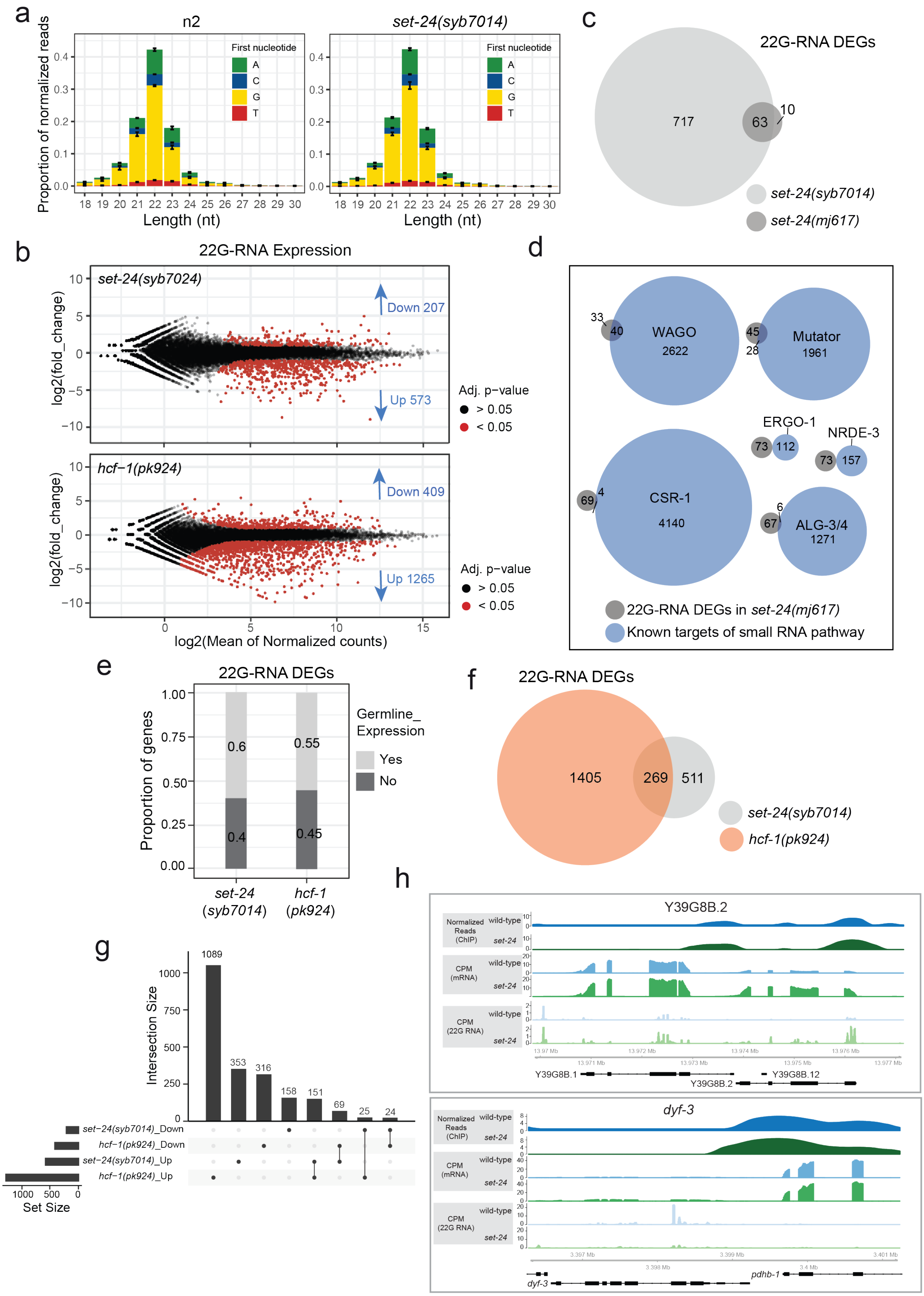

a
n2
set-24(syb7014)
Proportion of normalized reads
First nucleotide
A
C
G
T
Length (nt)
b
22G-RNA Expression
set-24(syb7024)
Down 207
Up 573
Adj. p−value
> 0.05
< 0.05
hcf−1(pk924)
Down 409
Up 1265
log2(fold_change)
log2(Mean of Normalized counts)
c
22G-RNA DEGs
717
63
10
set-24(syb7014)
set-24(mj617)
d
WAGO 2622
Mutator 1961
ERGO-1
NRDE-3
CSR-1 4140
ALG-3/4 1271
22G-RNA DEGs in set-24(mj617)
Known targets of small RNA pathway
e
22G-RNA DEGs
Proportion of genes
Germline_ Expression
Yes
No
set-24 (syb7014)
hcf-1 (pk924)
f
22G-RNA DEGs
1405
269
511
set-24(syb7014)
hcf-1(pk924)
g
Intersection Size
set−24(syb7014)_Down
hcf−1(pk924)_Down
set−24(syb7014)_Up
hcf−1(pk924)_Up
Set Size
h
Y39G8B.2
Normalized Reads (ChIP)
CPM (mRNA)
CPM (22G RNA)
wild-type
set-24
Y39G8B.1
Y39G8B.12
dyf-3
pdhb-1

Supplementary Fig. 11. Related to Figure 7. **SET-24 and HCF-1 regulate 22G-RNAs.**

a Plots showing the proportion of small RNAs beginning with different nucleotides in wild-type and *set-24(syb7014)* samples, which were treated with RppH. Small RNA-seq on *set-24(syb7014)* and wild-type, *n* = 4 independent replicates. **b** MA plots showing changes in 22G-RNA expression in *set-24(syb7014)* and *hcf-1(pk924)* worms. Statistical significance was assessed using a two-sided unpaired *t*-test. Small RNA-seq of *set-24(syb7014)*, *hcf-1(pk924)* and their respective wild-type controls, *n* = 4 independent replicates. **c** Venn diagram showing overlap of targets with differentially expressed 22G-RNAs in *set-24(syb7014) and set-24(mj617)* (fold change > 2 and 5% FDR). Small RNA-seq of *set-24(syb7014)*, *set-24(mj617),* and their respective wild-type controls, *n* = 4 independent replicates. **d** Venn diagrams showing overlaps between lists of genes with deregulated 22G-RNA levels in *set-24(mj617)* mutants (fold change > 2 and 5% FDR) and genes targeted by specific small RNA pathways. **e** Germline-expression analysis of targets of differentially expressed 22G-RNAs in *set-24(syb7014) and hcf-1(pk924).* **f** Venn diagrams showing overlap of targets of differentially expressed 22G-RNAs in *set-24(syb7014) and hcf-1(pk924)* (fold change > 2 and 5% FDR). **g** UpSet plot showing overlap of targets of up- and down- regulated 22G-RNAs in *set-24(syb7014) and hcf-1(pk924)*. **h** H3K4me3 enrichment, mRNA, and 22G-RNA levels of the selected genes (Y39G8B.2 and *dyf-3*) in wild-type and *set-24*(*syb7014*) mutant strains.
