# Supplementary material for: A SET domain-containing protein and HCF-1 maintain transgenerational epigenetic memory
Source: Nat Commun. 2026 Jan 9;17:1462. doi: 10.1038/s41467-025-68200-7 (PMC12886896; doi:10.1038/s41467-025-68200-7)
Supplement: Supplementary file 2 — Description of Additional Supplementary Files [file 41467_2025_68200_MOESM2_ESM.pdf]

Description of Additional Supplementary Files

File name: **Supplementary Data 1**.
Description: Immunoprecipitation-Mass spectrometry results of anti-FLAG IPs on N2 (expN) and SET-24::3xFLAG (expS) young adult worms.

File name: **Supplementary Data 2.**
Description: Yeast Two-Hybrid screening results of SET-24 protein.

File name: **Supplementary Data 3.**
Description: Log2fold-change of mRNAs in *set-24(syb7014)* compared to wild-type.

File name: **Supplementary Data 4.**
Description: Lists of H3K4me3 enriched genes with upregulated mRNAs, upregulated 22G-RNA targets or downregulated 22G-RNA targets in *set-24(syb7014)*.

File name: **Supplementary Data 5.**
Description: WormExp enrichment analysis of H3K4me3 enriched genes with upregulated mRNAs, upregulated 22G-RNA targets, or downregulated 22G-RNA targets.

File name: **Supplementary Data 6.**
Description: Log2fold-change of 22G-RNAs in mutants compared to wild-type.

File name: **Supplementary Data 7.**
Description: Lists of overlapping targets between SET-24-regulated 22G-RNA targets and WAGO- or Mutator- dependent targets.

File name: **Supplementary Data 8.**
Description: Worm strains used in this work.

File name: **Supplementary Data 9.**
Description: qPCR primers used in this work.
