# Supplementary material for: A SET domain-containing protein and HCF-1 maintain transgenerational epigenetic memory
Source: Nat Commun. 2026 Jan 9;17:1462. doi: 10.1038/s41467-025-68200-7 (PMC12886896; doi:10.1038/s41467-025-68200-7)
Supplement: Supplementary file 12 — Reporting Summary [file 41467_2025_68200_MOESM12_ESM.pdf]

nature portfolio

Corresponding author(s): Eric A. Miska
Last updated by author(s): 11/23/2025

Reporting Summary

Nature Portfolio wishes to improve the reproducibility of the work that we publish. This form provides structure for consistency and transparency in reporting. For further information on Nature Portfolio policies, see our Editorial Policies and the Editorial Policy Checklist.

Statistics

For all statistical analyses, confirm that the following items are present in the figure legend, table legend, main text, or Methods section.

n/a	Confirmed	
☐	☒	The exact sample size (*n*) for each experimental group/condition, given as a discrete number and unit of measurement
☐	☒	A statement on whether measurements were taken from distinct samples or whether the same sample was measured repeatedly
☐	☒	The statistical test(s) used AND whether they are one- or two-sided<br>*Only common tests should be described solely by name; describe more complex techniques in the Methods section.*
☒	☐	A description of all covariates tested
☒	☐	A description of any assumptions or corrections, such as tests of normality and adjustment for multiple comparisons
☐	☒	A full description of the statistical parameters including central tendency (e.g. means) or other basic estimates (e.g. regression coefficient) AND variation (e.g. standard deviation) or associated estimates of uncertainty (e.g. confidence intervals)
☐	☒	For null hypothesis testing, the test statistic (e.g. *F*, *t*, *r*) with confidence intervals, effect sizes, degrees of freedom and *P* value noted<br>*Give P values as exact values whenever suitable.*
☒	☐	For Bayesian analysis, information on the choice of priors and Markov chain Monte Carlo settings
☒	☐	For hierarchical and complex designs, identification of the appropriate level for tests and full reporting of outcomes
☐	☒	Estimates of effect sizes (e.g. Cohen's *d*, Pearson's *r*), indicating how they were calculated

Our web collection on statistics for biologists contains articles on many of the points above.

Software and code

Policy information about availability of computer code

Data collection	Images were taken by a Leica SP8 fluorescence microscope or a Nikon Ti2-E inverted microscope (NIS-Elements AR v5.42.06).
Data analysis	High-throughput seqencing analysis used RStudio (version 2022.07.2).<br>The protein sequence alignment was input to infer a maximum likelihood phylogenetic tree with IQ TREE v2.1.2.

For manuscripts utilizing custom algorithms or software that are central to the research but not yet described in published literature, software must be made available to editors and reviewers. We strongly encourage code deposition in a community repository (e.g. GitHub). See the Nature Portfolio guidelines for submitting code & software for further information.

Data

Policy information about availability of data

All manuscripts must include a data availability statement. This statement should provide the following information, where applicable:

- Accession codes, unique identifiers, or web links for publicly available datasets
- A description of any restrictions on data availability
- For clinical datasets or third party data, please ensure that the statement adheres to our policy

Sequencing data have been deposited to the NCBI Gene Expression Omnibus (GEO), and proteomics data are available at the ProteomeXchange Consortium via PRIDE. GEO: GSE291568, PRIDE: PXD057349.

Research involving human participants, their data, or biological material

Policy information about studies with human participants or human data. See also policy information about sex, gender (identity/presentation), and sexual orientation and race, ethnicity and racism.

Reporting on sex and gender	This information has not been collected.
Reporting on race, ethnicity, or other socially relevant groupings	See above.
Population characteristics	See above.
Recruitment	See above.
Ethics oversight	See above.

Note that full information on the approval of the study protocol must also be provided in the manuscript.

Field-specific reporting

Please select the one below that is the best fit for your research. If you are not sure, read the appropriate sections before making your selection.

☒ Life sciences ☐ Behavioural & social sciences ☐ Ecological, evolutionary & environmental sciences

For a reference copy of the document with all sections, see nature.com/documents/nr-reporting-summary-flat.pdf

Life sciences study design

All studies must disclose on these points even when the disclosure is negative.

Sample size	For experiments involving imaging and quantification, numbers of animals were > =10.
Data exclusions	No data were excluded from the analysis.
Replication	Phenotype experiments were repeated at least 3 times.
Randomization	Animals used in the experiments were cultured simultaneously on the same plates and selected randomly to control or experimental groups.
Blinding	The investigators were blinded to group allocation during data collection and analysis.

Reporting for specific materials, systems and methods

We require information from authors about some types of materials, experimental systems and methods used in many studies. Here, indicate whether each material, system or method listed is relevant to your study. If you are not sure if a list item applies to your research, read the appropriate section before selecting a response.

Materials & experimental systems

n/a	Involved in the study	
☐	☒	Antibodies
☒	☐	Eukaryotic cell lines
☒	☐	Palaeontology and archaeology
☐	☒	Animals and other organisms
☒	☐	Clinical data
☒	☐	Dual use research of concern
☒	☐	Plants

Methods

n/a	Involved in the study	
☐	☒	ChIP-seq
☒	☐	Flow cytometry
☒	☐	MRI-based neuroimaging

Antibodies

Antibodies used	The primary antibodies used were β-actin (Beyotime, AF5003), H3 (Abcam, ab1791), H3K4me2 (Abcam, ab7766), H3K4me3 (Abcam, ab8580), H3K4me3(Active Motif, #39159), H3K9me1 (Abcam, ab9045), H3K9me2 (Abcam, ab1220), H3K9me3 (Millipore, 07-523), H3K23me2 (Active Motif, 39653), H3K23me3 (Active Motif, 61499), H3K27me3 (Millipore, 07-449), H3K36me3 (Abcam, ab9050), H3K27Ac (Abcam, ab4729), H3PanAc (Abcam, ab47915), H4PanAc (Abcam, ab177790), H4 (Abcam, ab10158), Tubulin (Abcam,

	ab6160), FLAG (Sigma, F1804), GFP (Abcam, ab290), GST (Abcam, ab9085), MBP (Abcam, ab49923; Abcam, ab9084). The secondary antibodies used were goat anti-mouse (Beyotime, A0216; Abcam, ab6789), goat anti-rabbit (Abcam, ab205718; Abcam, ab6721), and goat anti-rat (Abcam, ab6734).
Validation	The primary antibodies have been validated by the manufactures. (See the website of manufactures)

Animals and other research organisms

Policy information about studies involving animals; ARRIVE guidelines recommended for reporting animal research, and Sex and Gender in Research

Laboratory animals	The Bristol strain N2 was used as the standard wild-type strain. All strains were grown at 20°C unless otherwise specified. The strains used in this study are listed in Supplementary table 8.
Wild animals	No wild animals were used in this study.
Reporting on sex	Hermaphrodite animals were used in this study.
Field-collected samples	No field-collected samples were used in this study.
Ethics oversight	Caenorhabditis elegans was used in this study and no ethical approval was required.

Note that full information on the approval of the study protocol must also be provided in the manuscript.

Plants

Seed stocks	No plant seed stocks were involved in this study.
Novel plant genotypes	No plant was involved in this study.
Authentication	No plant was involved in this study.

ChIP-seq

Data deposition

☒ Confirm that both raw and final processed data have been deposited in a public database such as GEO.

☒ Confirm that you have deposited or provided access to graph files (e.g. BED files) for the called peaks.

Data access links<br>*May remain private before publication.*	GEO: GSE291568<br>https://www.ncbi.nlm.nih.gov/geo/query/acc.cgi?acc=GSE291568
Files in database submission	n2_input_1<br>n2_input_2<br>SX3735_input_1<br>SX3735_input_2<br>n2_H3K4me3_1<br>n2_H3K4me3_2<br>SX3735_H3K4me3_1<br>SX3735_H3K4me3_2<br>n2_H3_1<br>n2_H3_2<br>n2_H3_3<br>SX3735_H3_1<br>SX3735_H3_2<br>SX3735_H3_3
Genome browser session<br>(e.g. UCSC)	WBcel235<br>IGV_2.18.4

Methodology

Replicates	Two replicates for H3K4me3 ChIP-se in n2 and SX3735.

Sequencing depth	20 million paired-end reads were sequenced for every ChIP yield DNA product. Over 85% reads were clean reads (Not adapter related). 2374 H3K4me3 peaks from n2 and 3079 peaks from SX3735 were called.
Antibodies	H3K4me3 antibody, Active Motif, #39159; H3 antibody, Abcam, #ab1791
Peak calling parameters	Peak calling was performed using Macs3 CallPeak (v3.0.0b1) with no cutoff (-q 1) and the options --extsize 200 and --nomodel.
Data quality	2374 H3K4me3 peaks from n2 and 3079 peaks from SX3735 were at FDR<5%.
Software	Adapter sequences were removed using Cutadapt 1.18 in the pair-ended read mode. Reads were then mapped to the C. elegans genome (WBcel235) using the Burrows-Wheeler Aligner with the MEM algorithm (BWA 0.7.17-r1188). Mapped reads were indexed and sorted using Samtools 1.10. Bam files were filtered with samtools to remove non-unique mappers, secondary alignments and low-quality pairs (MAPQ<10). Duplicate reads were removed using Picard MarkDuplicates 3.1.0-3 with the --REMOVE_DUPLICATES option. Peak calling was performed using Macs3 CallPeak (v3.0.0b1) with no cutoff (-q 1) and the options --extsize 200 and --nomodel. Differential binding analysis was conducted using DiffBind (v3.12.0) in R (v4.3.3) over a 200 bp sliding window with 10 bp shift across the entire genome. Counts were obtained without merging overlapping peaks and without computing summits. Normalization was performed using the DESeq2 method, accounting for library size, and differential analysis followed DiffBind's DESeq2 implementation.
